# Fecal microbiota composition is related to brown adipose tissue ^18^F-fluorodeoxyglucose uptake in young adults

**DOI:** 10.1007/s40618-022-01936-x

**Published:** 2022-10-15

**Authors:** L. Ortiz-Alvarez, F. M. Acosta, H. Xu, G. Sanchez-Delgado, R. Vilchez-Vargas, A. Link, J. Plaza-Díaz, J. M. Llamas, A. Gil, I. Labayen, P. C. N. Rensen, J. R. Ruiz, B. Martinez-Tellez

**Affiliations:** 1 PROFITH (PROmoting FITness and Health Through Physical Activity) Research Group, Sport and Health University Research Institute (iMUDS), University of Granada, Granada, Spain; 2 Department of Biochemistry and Molecular Biology II, School of Pharmacy, University of Granada, Granada, Spain; 3 Turku PET Centre, University of Turku, Turku, Finland; 4 Turku PET Centre, Turku University Hospital, Turku, Finland; 5 InFLAMES Research Flagship Centre, University of Turku, Turku, Finland; 6 Department of Physical and Sports Education, School of Sports Science, University of Granada, Granada, Spain; 7 Pennington Biomedical Research Center, Baton Rouge, LA 70808 USA; 8 Department of Gastroenterology, Hepatology and Infectious Diseases, Otto-Von-Guericke-University Magdeburg, Magdeburg, Germany; 9 Children’s Hospital of Eastern Ontario Research Institute, Ottawa, ON K1H 8L1 Canada; 10 Instituto de Investigación Biosanitaria Ibs Granada, 18014 Granada, Spain; 11 Servicio de Medicina Nuclear, Hospital Universitario Virgen de las Nieves, Granada, Spain; 12 Centro de Investigación Biomédica En Red (CIBER) Fisiopatología de la Obesidad y Nutrición (CIBEROBN), Instituto de Salud Carlos III (ISCIII), Málaga, Spain; 13 Institute of Nutrition and Food Technology “José Mataix”, Biomedical Research Center, Parque Tecnológico Ciencias de la Salud, University of Granada, Armilla, Granada, Spain; 14 Instituto de Investigación Biosanitaria, Ibs.Granada, Granada, Spain; 15 Institute for Innovation and Sustainable Development in Food Chain (IS-FOOD), Public University of Navarra, Campus de Arrosadía, Pamplona, Spain; 16 Department of Medicine, Division of Endocrinology, and Einthoven Laboratory for Experimental Vascular Medicine, Leiden University Medical Center, Leiden, The Netherlands; 17 Department of Education, Faculty of Education Sciences, SPORT Research Group (CTS-1024), CERNEP Research Center, University of Almería, Almería, Spain

**Keywords:** Brown fat, Glucose uptake, Gut microbiota, Obesity, Short-chain fatty acids

## Abstract

**Objective:**

Human brown adipose tissue (BAT) has gained considerable attention as a potential therapeutic target for obesity and its related cardiometabolic diseases; however, whether the gut microbiota might be an efficient stimulus to activate BAT metabolism remains to be ascertained. We aimed to investigate the association of fecal microbiota composition with BAT volume and activity and mean radiodensity in young adults.

**Methods:**

82 young adults (58 women, 21.8 ± 2.2 years old) participated in this cross-sectional study. DNA was extracted from fecal samples and 16S rRNA sequencing was performed to analyse the fecal microbiota composition. BAT was determined via a static ^18^F-fluorodeoxyglucose (^18^F-FDG) positron emission tomography-computed tomography scan (PET/CT) after a 2 h personalized cooling protocol. ^18^F-FDG uptake was also quantified in white adipose tissue (WAT) and skeletal muscles.

**Results:**

The relative abundance of *Akkermansia*, *Lachnospiraceae sp.* and *Ruminococcus* genera was negatively correlated with BAT volume, BAT SUVmean and BAT SUVpeak (all rho ≤ − 0.232, *P* ≤ 0.027), whereas the relative abundance of *Bifidobacterium* genus was positively correlated with BAT SUVmean and BAT SUVpeak (all rho ≥ 0.262, *P* ≤ 0.012). On the other hand, the relative abundance of *Sutterellaceae* and *Bifidobacteriaceae* families was positively correlated with ^18^F-FDG uptake by WAT and skeletal muscles (all rho ≥ 0.213, *P* ≤ 0.042). All the analyses were adjusted for the PET/CT scan date as a proxy of seasonality.

**Conclusion:**

Our results suggest that fecal microbiota composition is involved in the regulation of BAT and glucose uptake by other tissues in young adults. Further studies are needed to confirm these findings.

**Clinical trial information:**

ClinicalTrials.gov no. NCT02365129 (registered 18 February 2015).

**Supplementary Information:**

The online version contains supplementary material available at 10.1007/s40618-022-01936-x.

## Introduction

Brown adipose tissue (BAT) is a tissue that dissipates energy through the action of the uncoupling protein-1 (UCP1) in rodents and in humans [1]. Moreover, BAT takes up and oxidizes glucose and lipids, as such working as a nutrient sink, and through its endocrine function may have cardiometabolic benefits [2]. Consequently, BAT activation has been suggested as a potential therapeutic target to combat obesity and its related cardiometabolic diseases [3].

The human gut harbours a vast array of microorganisms such as Eukarya, Archaea, fungi, and mainly bacteria [4], commonly known as gut microbiota [5]. Bacteria are classified into five different phyla, of which in humans *Firmicutes* and *Bacteroidetes* are found in higher abundance (> 75%) compared to *Proteobacteria*, *Verrucomicrobia*, and *Actinobacteria* (< 25%) [6]. Although cold exposure is the main physiological activator of BAT [7], evidence suggests that gut microbiota is an important endogenous factor that can modulate BAT metabolism [8–12]. Indeed, gut microbiota composition can promote whole-body thermogenesis during cold exposure in mice, with BAT being the main thermogenic effector [11, 12]. Gut microbiota might be involved in the process of remodelling white adipose tissue (WAT) towards a beige-like phenotype in individuals with obesity [13–15]. However, a recent study showed that fecal microbiota composition was not associated with BAT activity after cold exposure in individuals with non-alcoholic fatty liver disease [16], which appears to be in contrast with previous evidence from rodent models [17–20]. Further research is therefore needed to understand the role of gut microbiota composition in human BAT metabolism.

We hypothesized that the relative abundance of gut bacteria previously reported to improve obesity and cardiometabolic diseases, such as *Akkermansia* [21] or *Bifidobacterium* [22] genera, is associated with BAT volume and activity. Thus, the main aim of the present study was to investigate the association of fecal microbiota composition with BAT volume and activity, as determined via cold-induced ^18^F-fluorodeoxyglucose (^18^F-FDG) uptake, and mean radiodensity in young adults. Additionally, we explored the association of fecal microbiota composition with the ^18^F-FDG uptake by white adipose tissue (WAT) and skeletal muscles.

## Material and methods

### Design study and participants

This cross-sectional study was carried out within the framework of the ACTIBATE study [23] (Clinical Trials.gov ID: NCT02365129). A total of 92 young healthy adults (27 men and 65 women, age: 18–25 years old) took part in this study. The assessments were performed in Granada (Spain) between October and November 2016. All participants underwent a comprehensive medical examination and reported themselves to be sedentary (<20 min moderate-vigorous physical activity on < 3 days/week), to have a stable body weight over the last 3 months (< 3 kg change), not to be exposed to cold regularly, neither be pregnant, smoking, or taking any regular medication (including antibiotics) that affects the cardiovascular system, or presenting any acute or chronic illness.

### Body composition assessment

We measured the participants’ weight and height while being barefoot and wearing light clothing, using a SECA scale and stadiometer (model 799, Electronic Column Scale, Hamburg, Germany). Lean body mass and body fat mass were determined by Dual Energy X-ray Absorptiometry (Hologic Discovery Wi, Marlborough, MA, USA). Body mass index (BMI), lean mass index and fat mass index were calculated as weight, lean body mass and body fat mass in kg divided by height in meters square (m^2^).

### Fecal microbiota composition analyses

#### Stool collection and DNA extraction

The participants collected a fecal sample (50–60 g) 3 ± 7 days [mean ± standard deviation] prior to the positron emission tomography/computed tomography (PET/CT). They transported the fecal sample in plastic sterile containers inside a portable cooler until arrival at the research centre. Fecal samples were stored at −80 °C until the extraction of deoxyribonucleic acid (DNA). A QIAamp DNA Stool Mini Kit (QIAGEN, Barcelona, Spain) was utilized to extract DNA following the manufacturer’s instructions, and samples were incubated at 95 ºC to ensure lysis of both Gram-positive and Gram-negative bacteria. The quantification of DNA was performed using a NanoDrop ND1000 spectrophotometer (Thermo Fisher Scientific, DE, USA). We measured absorbance spectrophotometrically at A260/280 nm and A260/230 nm ratios for determining DNA purity. The A260/280 ratio is used to determine protein contamination [24], whereas the A260/230 ratio indicates the presence of organic contaminants (salt and phenol) in nucleic acid samples [25].

#### Sequencing analysis

We amplified DNA extracted by polymerase chain reaction (PCR) with primer pairs, 16S Amplicon PCR Forward Primer: 5’CCTACGGGNGGCWGCAG, and 16S Amplicon PCR Reverse Primer: 5’GACTACHVGGGTATCTAATCC targeting the V3 and V4 hypervariable regions of the bacterial 16S rRNA gene [26]. All PCRs were executed in 25 µL reaction volumes incorporating 12.5 µL 2X KAPA HiFi Hotstart ready mix (KAPA Biosystems, Woburn, MA, USA), 5 µL of each forward and reverse primers (1 µM) and 2.5 µL of extracted DNA (10 ng) following denaturation at 95 ºC for 3 min, 8 cycles of denaturation at 95 ºC for 30 s, annealing at 55 ºC for 30 s, elongation at 72 ºC for 30 s, and a final extension at 72 ºC for 5 min. PCR clean-up was executed using AMPure XP beads (Beckman Coulter, Indianapolis, IN, USA) to purify the 16S V3 and V4 amplicon from free primers and primer dimer species. The next step was the PCR index (same condition that before); in this step, we used a Nextera XT index kit (Illumina, San Diego, CA, USA) to tag DNA with sequencing adapters. AMPure XP beads (Beckman Coulter, Indianapolis, IN, USA) were used for purifying the pooled PCR products before quantification. The consequential amplicons were sequenced at MiSeq (Illumina, USA), using a paired-end (2 × 300nt) Illumina MiSeq sequencing system (Illumina, San Diego, CA, USA).

#### Bioinformatics analyses

“Dada2” [27] R [28] package was used for analysing the generated FastQ files, which retrieved 11,659,014 paired-end with an average of 127 ± 33 × 10^3^ reads per sample. All samples surpassed a cut-off of 10,000 reads. Samples were standardized to equal size of 30,982 reads using “phyloseq” [29] R [28] package, retrieving 11,158 phylotypes.

Taxonomic affiliation of phylotypes was assigned using the “classifier” function from Ribosomal Database Project (RDP), based on the naive Bayesian classification [30] with a pseudo-bootstrap threshold of 80%. We obtained a total of 209 genera that belong to 16 different phyla. Bacterial communities were analysed at different taxonomic levels (phylum to genera), calculating relative abundances in each sample as “(nº reads/total nº reads) × 100 per sample” [31], and expressed as percentages. The analyses were performed using the taxonomic levels with more than 0.5% of the relative abundance of average between samples.

### Brown adipose tissue measurements

#### Shivering threshold test

To personalize the cooling protocol used to activate human BAT, subjects underwent a cooling test 48–72 h before the ^18^F-FDG PET/CT scan, in which their shivering threshold was determined. Briefly, participants arrived in fasted condition (≥ 6 h), having avoided alcoholic or stimulant beverages within the last 12 h, and having refrained from any moderate physical activity within the last 24 h, and vigorous activity within the last 48 h. They wore standardized clothes (sandals, T-shirt and shorts) and entered a warm room (22.1 ± 1.6 ºC) where they kept seated for 30 min. Later, they entered a cool room (19.8 ± 0.5 °C) and sat down in a chair, where we put them a water-perfused cooling vest (Polar Products Inc., Stow, OH, USA). Each participant’s shivering threshold was then determined being seated and following a personalized cooling protocol, as described elsewhere [32, 33]. Water temperature started at 16.6 °C and decreased ~ 1.4 °C every 10 min until shivering occurred (self-reported and visually observed by the researchers). The water temperature at which shivering occurred was considered the shivering threshold and used for determining the cooling vest water temperature during the personalized cooling protocol before the ^18^F-FDG-PET/CT scan (4 °C above the shivering threshold) [32].

#### Personalized cooling protocol prior to positron emission tomography/computed tomography scan

After 48–72 h of the shivering threshold test, the participants went to the *Hospital Virgen de las Nieves*, Granada (Spain) for assessment of BAT volume, activity and mean radiodensity. They rested in a warm room for 30 min and then they entered a cool room (19.5–20.0 °C), where they dressed with the same cooling vest. On this occasion, the water temperature was set ~ 4 °C above their shivering threshold. After the first hour of cold exposure, the participants received an intravenous injection of ~ 185 MBq ^18^F-FDG while the water temperature was increased by 1 °C to prevent shivering. One hour after the injection, the participants underwent a static PET/CT scan, using a Siemens Biograph 16 PET/CT scanner (Siemens, Erlangen, Germany), scanning from the atlas to approximately the mid-chest. The date when PET/CT scan was performed was recorded as the day of the year, being January 1st day 1, and December 31st day 366. This date was used as a proxy of seasonal variation.

#### Brown adipose tissue quantification

All PET/CT images were examined using the Beth Israel plug-in for FIJI software (http://sourceforge.net/projects/bifijiplugins) [34], following a protocol described elsewhere [32, 33] and in agreement with current methodological recommendations (BARCIST 1.0) [35]. To determine BAT volume and mean and peak standardized uptake values (SUVmean and SUVpeak), six regions of interest (ROIs) were outlined from the atlas vertebra to thoracic vertebra 4 using a 3D-axial technique. These ROIs comprised the supraclavicular, laterocervical, paravertebral and mediastinal regions. Those voxels with a radiodensity between −190 and −10 Hounsfield Units (HU) and a SUV higher than the individualized SUV threshold, calculated as 1.2/(lean body mass/body mass), were classified as BAT voxels [35]. BAT volume was computed as the sum of the volume of these voxels across all ROIs. BAT SUVmean was determined as the average SUV of all voxels, and SUVpeak as the average of the three voxels presenting the highest SUV within 1 cm^3^ from the voxel presenting the highest SUV, and meeting the above-mentioned criteria, across all ROIs. All SUV values were expressed relative to lean body mass [36]. BAT mean radiodensity was calculated as the average radiodensity of those voxels meeting the aforementioned criteria in a single region of interest covering the whole body from the atlas to thoracic vertebrae 4, except the mouth. Additionally, we determined the descending aorta (used as a reference tissue) SUVpeak. This was done by drawing a one-slice ROI in the descending aorta, at the height of thoracic vertebra 4. We also determined the SUVpeak in the tricipital WAT [37], as well as in different skeletal muscles, including the cervical, scalene, longus colli, paravertebral, subscapular, sternocleidomastoid, supraspinatus, trapezius, deltoid, pectoralis major, and triceps brachii muscles on both the right and left sides of the body [37]. Then, we obtained the average for the SUVpeak values of all examined muscles to provide a representative value for all skeletal muscle ^18^F-FDG uptake.

### Statistical analysis

Data are presented as means ± standard deviations unless otherwise stated. All variables were tested for normality using D’Agostino and Pearson omnibus. Most of the variables displayed a non-normal distribution and, thus, non-parametric tests were used for all analyses. We did not detect any sex interaction across the variables studied (Fig. S1 and Table S1), nor differences between the status of BMI (data not shown); therefore, all main data for men and women, as well as the status of BMI, were pooled together. Partial Spearman correlations were used to investigate the correlation of fecal microbiota composition with BAT volume, ^18^F-FDG uptake and mean radiodensity by “psych” [38] and “corrplot” [39] R [28] packages. Since seasonality could affect measurements of BAT [40–43], we presented all the analyses adjusting for the PET/CT scan date, that is the natural day. The level of significance was set at *P* < 0.05. SPSS (SPSS v. 22.0, IBM SPSS Statistics, IBM Corp., Armonk, NY, USA), R software (V.3.6.0; http://www.R-project.orghttp://www.R-project.org) [28], and GraphPad Prism version 8.0.0 for Windows (GraphPad Software, San Diego, California, USA) were used for the statistical analysis and graphical plots.

## Results

### Characteristics of participants

Among the 92 participants with a complete analysis of fecal microbiota composition, only 82 participants had BAT measurements; therefore, these 82 participants were finally included in the analyses. Table 1 shows the descriptive characteristics of those 82 participants, of whom 70.7% were women. Participants were 21.8 ± 2.2 years old, and their BMI was 24.9 ± 4.8 kg/m^2^.

**Table 1:** Descriptive characteristics of the participants

	*N*	Mean ± SD
Sex (women %)	82 (70.7%)	
Age (years)	82	21.8 ± 2.2
Body composition variables		
Body mass index (kg/m^2^)	82	24.9 ± 4.8
Lean mass index (kg/m^2^)	76	14.4 ± 2.3
Fat mass index (kg/m^2^)	76	8.9 ± 3.1
Fat mass percentage (%)	76	36.1 ± 7.9
PET/CT variables		
BAT volume (mL)	82	69.0 ± 59.9
BAT SUVmean	82	2.2 ± 1.0
BAT SUVpeak	82	6.4 ± 4.7
BAT Mean radiodensity (HU)	62	− 59.0 ± 9.7
Descending aorta SUVpeak	82	0.9 ± 0.2
Subcutaneous WAT Triceps SUVpeak	82	0.10 ± 0.05
All skeletal muscle SUVpeak	82	0.8 ± 0.2
Fecal microbiota variables		
*Composition (Phylum)*		
*Actinobacteria* (%)	82	1.7 ± 1.6
*Bacteroidetes* (%)	82	39.9 ± 8.9
*Firmicutes* (%)	82	48.3 ± 9.9
*Proteobacteria* (%)	82	6.7 ± 5.4
*Verrucomicrobia* (%)	82	2.3 ± 4.3

Data are presented as means ± standard deviations (SD). All SUV variables are shown relative to lean body mass

*BAT* brown adipose tissue, *HU* Hounsfield Units, *SUV* standardized uptake value, *WAT* white adipose tissue

### Relationship between fecal microbiota composition and cold-induced BAT variables

**Fig. 1:**
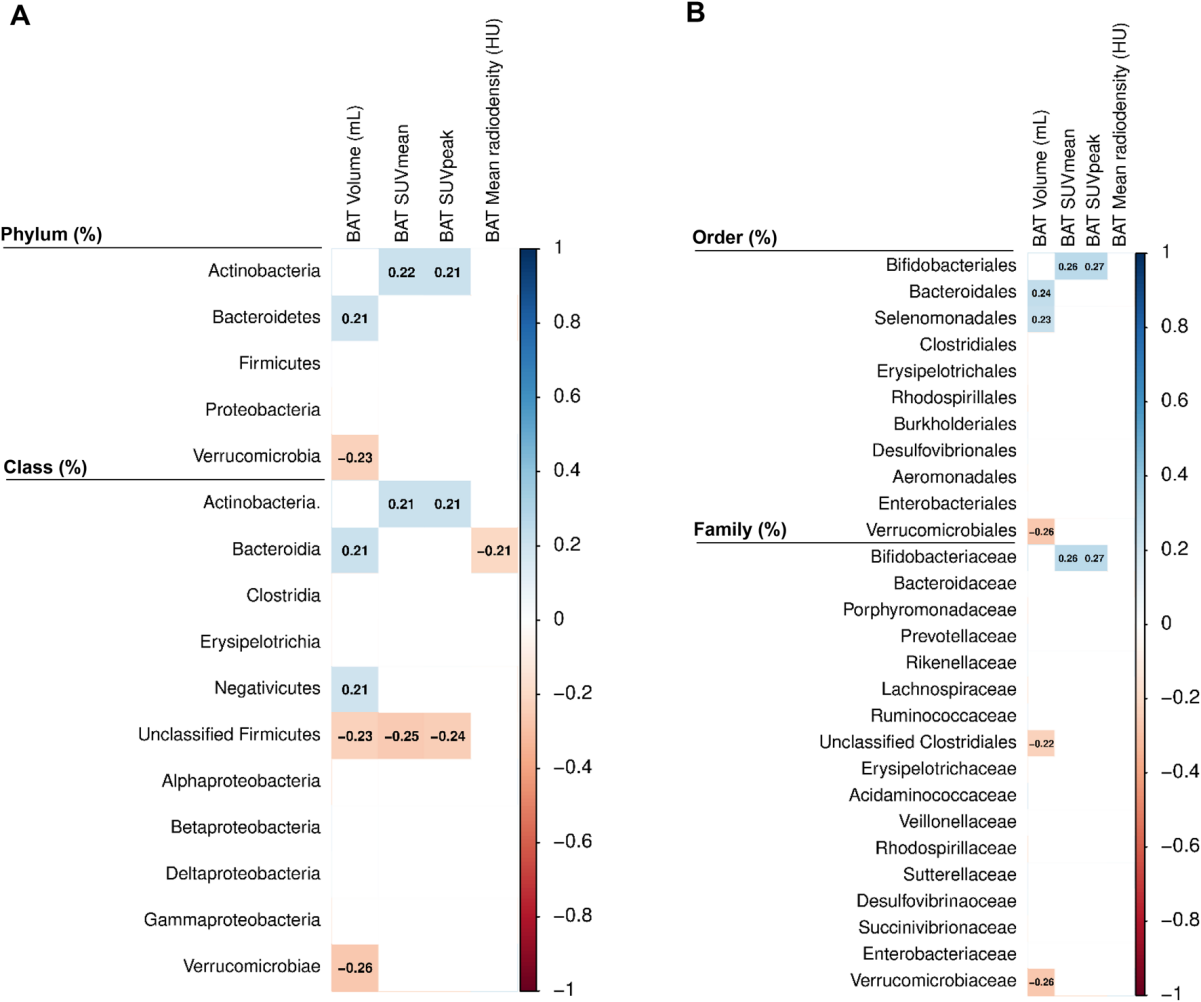
Partial Spearman correlation of fecal microbiota composition with BAT volume, SUVmean, SUVpeak, and mean radiodensity adjusted for the PET/CT scan date. Boxes represent the statistically significant (*P* < 0.05) correlations and the value within the boxes shows the partial Spearman correlation coefficient. Blue boxes indicate a positive correlation, whereas red boxes indicate a negative correlation between fecal microbiota composition with cold-induced BAT variables. Panel A shows phylum and class taxonomic levels and panel B indicates order and family taxonomic levels. BAT SUVmean and SUVpeak are shown relative to lean body mass. *BAT* brown adipose tissue, *HU* Hounsfield Units, *PET/CT* positron emission tomography/computed tomography, *SUV* standardized uptake value

We observed that the relative abundance of the *Verrucomicrobia* phylum and its lower taxonomic levels (class, order, and family) were negatively correlated with BAT volume (all rho ≤ − 0.229, *P* ≤ 0.029; Fig. 1). In contrast, the relative abundance of the *Actinobacteria* phylum and at lower taxonomic levels (class, order, and family) was positively correlated with BAT SUVmean (all rho ≥ 0.211, *P* ≤ 0.044; Fig. 1) and BAT SUVpeak (all rho ≥ 0.211, *P* ≤ 0.045; Fig. 1).

**Fig. 2:**
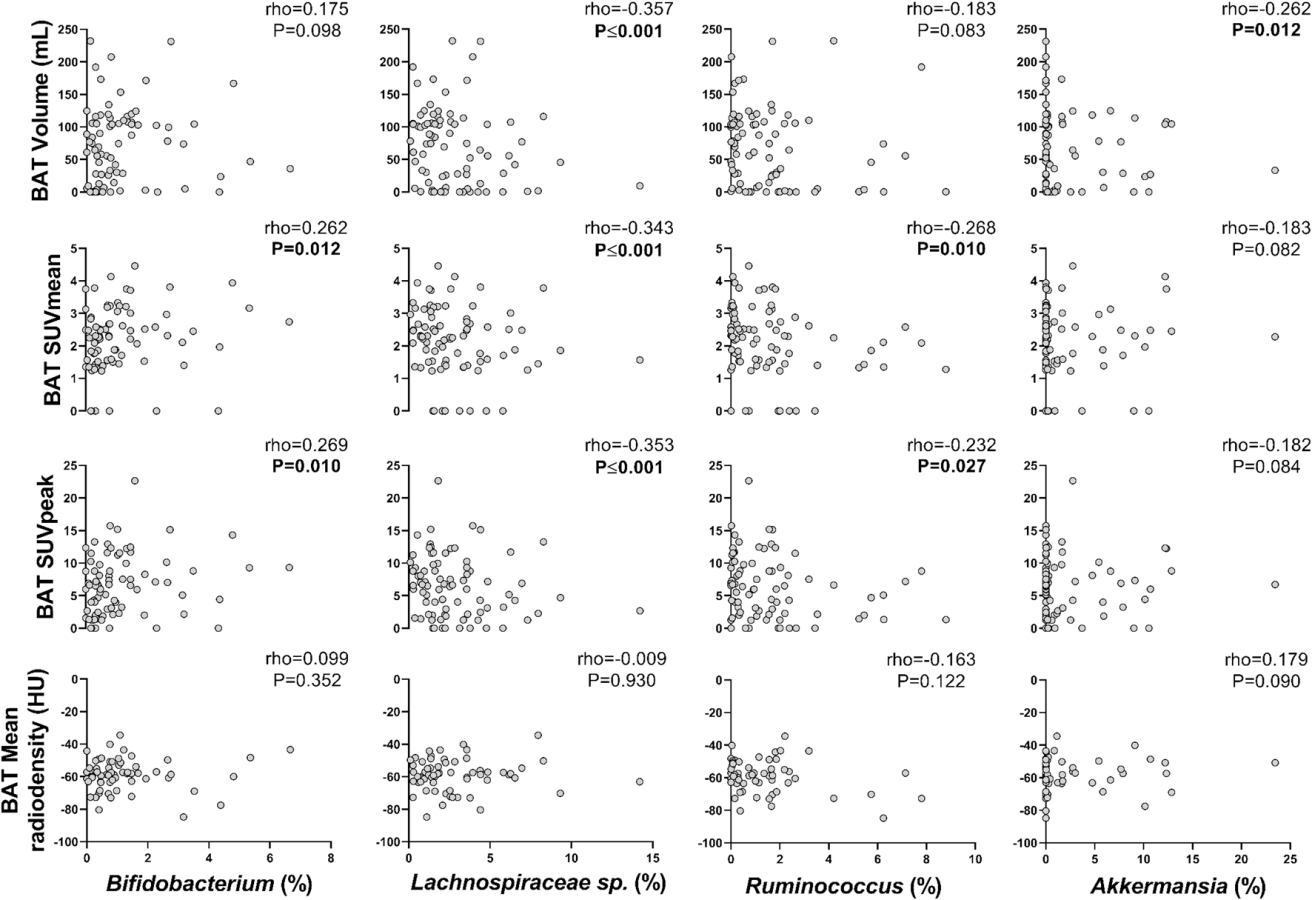
Partial Spearman correlations of relative abundance of *Bifidobacterium*, *Lachnospiraceae sp.*, *Ruminococcus* and *Akkermansia* genera with BAT volume, SUVmean, SUVpeak, and mean radiodensity, after adjusting for the PET/CT scan date. Rho = Partial Spearman's correlations coefficient. *P* = *p*-value from univariate partial Spearman correlation. BAT SUVmean and SUVpeak are shown relative to lean body mass. *BAT* brown adipose tissue, *HU* Hounsfield Units, *PET/CT* positron emission tomography/computed tomography, *SUV* standardized uptake value

Next, we investigated whether the relative abundance of specific genera within the above-mentioned taxonomic groups was associated with BAT-related variables (Fig. 1). We found that the relative abundance of *Akkermansia* genus (*Verrucomicrobia* phylum) was negatively correlated with BAT volume (rho = − 0.262, *P* = 0.012; Fig. 2), whereas the relative abundance of *Bifidobacterium* genus (*Actinobacteria* phylum) was positively correlated with BAT SUVmean (rho = 0.262, *P* = 0.012; Fig. 2) and BAT SUVpeak (rho = 0.269, *P* = 0.010; Fig. 2). Moreover, the relative abundance of *Lachnospiraceae sp.* and *Ruminococcus* genera (both from *Firmicutes* phylum) was negatively correlated with BAT SUVmean (rho ≤ -0.268, *P* ≤ 0.010; Fig. 2) and BAT SUVpeak (rho ≤ -0.232, *P* ≤ 0.027; Fig. 2), although only the relative abundance of *Lachnospiraceae sp.* genus (*Firmicutes* phylum) was negatively correlated with BAT volume (rho = − 0.357, *P* ≤ 0.001; Fig. 2). All results persisted when the analyses were divided by sex (Fig. S1), and when participants with no detectable/scarce BAT glucose uptake were excluded from the analyses (data not shown).

### Relationship of fecal microbiota composition with cold-induced uptake of ^18^F-FDG by descending aorta, WAT and skeletal muscles

**Fig. 3:**
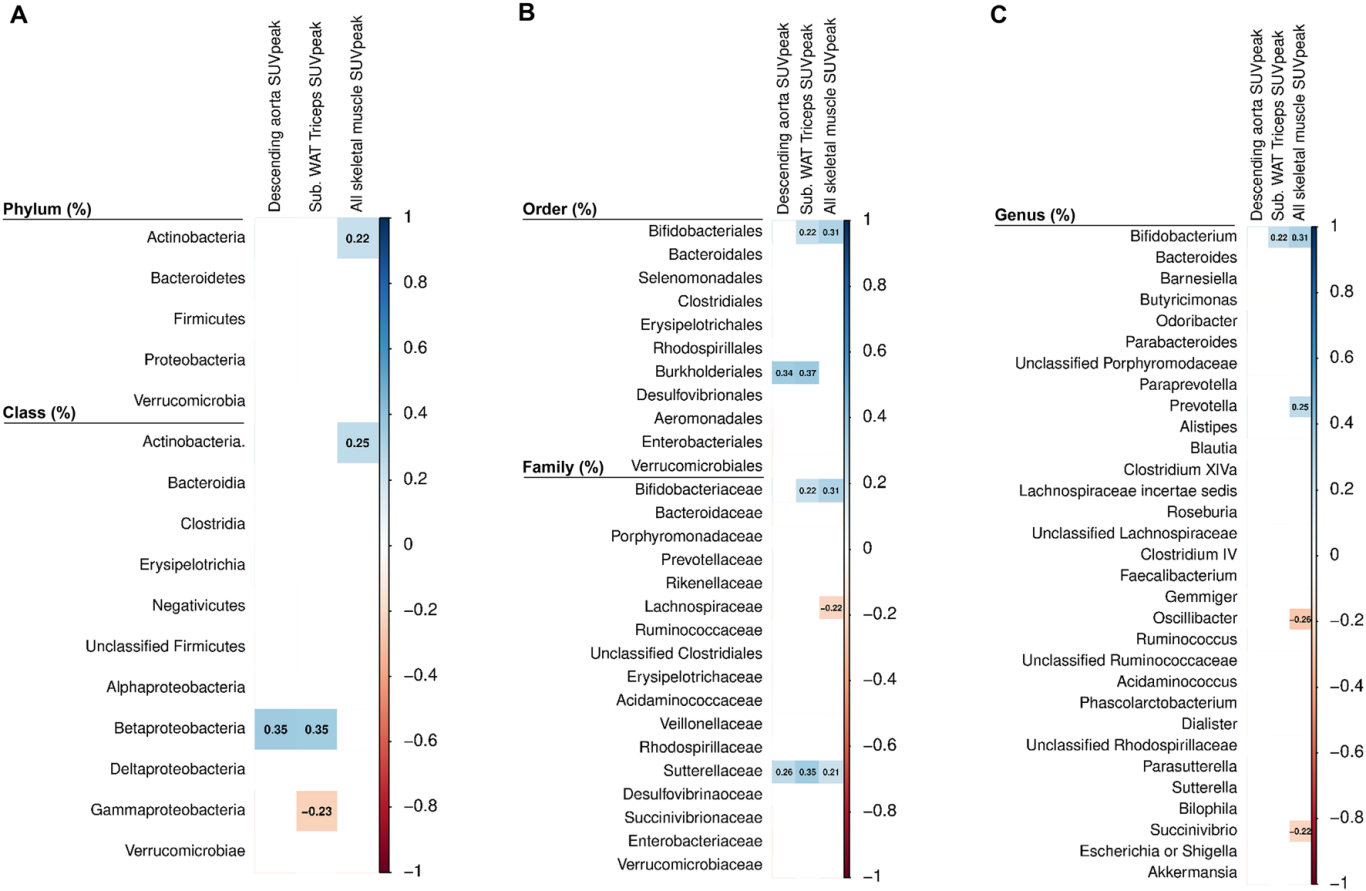
Partial Spearman correlation of fecal microbiota composition with tissues related to ^18^F-FDG uptake adjusted for the PET/CT scan date. Boxes only represent the statistically significant (*P* < 0.05) correlations and the value within the boxes shows the partial Spearman correlation coefficient. Blue boxes indicate a positive correlation whereas red boxes indicate a negative correlation between fecal microbiota composition with tissues related ^18^F-FDG uptake adjusted for PET/CT scan date. Panel A shows phylum and class taxonomic levels. Panel B indicates order and family taxonomic levels, and panel C shows genus taxonomic level. All SUV variables are shown relative to lean body mass. ^*18*^*F-FDG* [^18^F]fluorodeoxyglucose, *PET/CT* positron emission tomography/computed tomography, *SUV* standardized uptake value, *WAT* white adipose tissue

The relative abundance of *Betaproteobacteria* class, and at lower taxonomic levels (order and family; all *Proteobacteria* phylum) was positively correlated with the SUVpeak of the descending aorta (our reference tissue, all rho ≥ 0.257, *P* ≤ 0.014; Fig. 3) and the subcutaneous WAT triceps (all rho ≥ 0.347, *P* ≤ 0.001; Fig. 3), whereas the relative abundance of *Sutterellaceae* family (*Proteobacteria* phylum) was positively correlated with all skeletal muscles SUVpeak (rho = 0.213, *P* = 0.042; Fig. 3). In addition, the relative abundance of *Actinobacteria* phylum and its lower taxonomic levels (class, order, family and genus) were positively correlated with all skeletal muscles SUVpeak (all rho ≥ 0.225, *P* ≤ 0.032; Fig. 3), whereas only the *Bifidobacteriales* order and its lower taxonomic levels (family and genus) were positively correlated with subcutaneous WAT triceps SUVpeak (all rho ≥ 0.266, *P* ≤ 0.011; Fig. 3).

## Discussion

This study shows, for the first time, that the relative abundance of *Akkermansia, Lachnospiraceae sp.*, and *Ruminococcus* genera was negatively correlated with BAT volume and activity (as estimated by ^18^F-FDG uptake), whereas the relative abundance of *Bifidobacterium* genus was positively correlated with BAT activity. Moreover, *Bifidobacteriaceae* and *Sutterellaceae* families was positively correlated with ^18^F-FDG uptake by WAT in the tricipital area and skeletal muscles. These findings suggest that fecal microbiota composition is involved in glucose metabolism by BAT and other tissues including WAT and skeletal muscles in young adults.

The role of gut microbiota composition in BAT activation and metabolism has been investigated in mouse models [17–20], but studies in humans are scarce [16]. In fact, the only study in humans observed that the fecal microbiota composition was not associated with BAT activity after cold induction in individuals with non-alcoholic fatty liver disease [16]. The present study included a cohort of healthy young adults and found that the relative abundance of *Akkermansia, Lachnospiraceae sp.*, and *Ruminococcus* genera was negatively correlated with BAT volume and activity. It has been previously shown that the bacteria belonging to *Akkermansia* and *Ruminococcus* genera produce short-chain fatty acids (SCFAs), as acetate, to activate BAT thermogenesis and promote WAT browning via triggering the G-protein-coupled receptor (GPR) 43 in mice [17–20]. However, a recent study using single nuclei RNA sequencing in human BAT demonstrated that BAT is composed of a set of different subpopulations of adipocytes [44]. Indeed, they observed that a rare subpopulation of brown adipocytes inhibited the thermogenic capacity of neighbouring adipocytes via the production of acetate [44]. Accordingly, the same authors showed that local acetate induces BAT thermogenic dysfunction [45]. Therefore, evidence of the relationship between acetate and BAT activation is contradictory. Of note, our data appear to be in line with the former studies in human BAT [44, 45], showing that acetate-producing bacteria, such as the *Akkermansia, Lachnospiraceae sp.*, and *Ruminococcus* genera, are inversely correlated with BAT ^18^F-FDG uptake.

We also observed a positive correlation of the relative abundance of *Bifidobacteriaceae* and *Sutterellaceae* families with ^18^F-FDG uptake by WAT and skeletal muscles, whereas the relative abundance of *Bifidobacterium* genus, and at higher taxonomic levels, was positively correlated with BAT ^18^F-FDG uptake. Scientific evidence has shown that SCFAs produced by gut microbiota regulate glucose homeostasis and improve insulin sensitivity [46, 47]. This might be partially explained because SCFAs increase glucagon-like peptide 1 and 2 secretion from enteroendocrine L-cells by their binding to GPR41 and GPR43 [46, 47]. This fact leads to an increase in glucose uptake by metabolically active tissues [46, 47]. However, all these hypotheses should be confirmed in future experiments investigating whether these bacteria are actually able to increase BAT and other tissues’ glucose uptake.

### Limitations and strengths

The cross-sectional design of this study precluded us from establishing cause-effect relationships. Our study population includes young adults without relevant disease or comorbidity; thus, our results cannot be extrapolated to older or unhealthier populations. Importantly, SCFAs were not measured and could be of interest for future studies. Further, although BAT takes up glucose from circulation, intracellular fatty acids are the main substrate of brown adipocytes in humans [48]. Hence, ^18^F-FDG PET/CT might not estimate accurately the cold-induced BAT metabolic activity, despite being the most widely used technique. Moreover, it will be of scientific interest to investigate whether fecal microbiota composition is related to BAT activity measured at other room temperatures. As for the strengths of this study, we should stand out that we sequenced the microbiota composition using the latest technology (Illumina platform) and annotations were made with RDP until genera taxon.

### Conclusions

This is the first study showing a negative correlation of the relative abundance of *Akkermansia*, *Lachnospiraceae sp.* and *Ruminococcus* genera with cold-induced BAT volume and activity in young adults. Contrarily, the relative abundance of *Bifidobacterium* genus was positively correlated with BAT activity. Moreover, the relative abundance of *Bifidobacteriaceae* and *Sutterellaceae* families was positively correlated with ^18^F-FDG uptake by WAT and skeletal muscles. Altogether, these findings suggest that fecal microbiota is involved in the regulation of glucose uptake by human BAT and other metabolic tissues, but future studies are needed to confirm these findings and to elucidate underlying mechanisms.

## Supplementary Information

Below is the link to the electronic supplementary material.

Supplementary file1 (DOCX 523 KB)

Supplementary file2 (DOCX 16 KB)

## Data Availability

The datasets generated during the current study are available from the corresponding author upon reasonable request.
