# Supplementary material for: Fecal microbiota composition is related to brown adipose tissue 18F-fluorodeoxyglucose uptake in young adults
Source: J Endocrinol Invest. 2022 Oct 15;46(3):567–76. doi: 10.1007/s40618-022-01936-x (PMC9938059; doi:10.1007/s40618-022-01936-x)
Supplement: Supplementary file 1 — Supplementary file1 (DOCX 523 KB) [file 40618_2022_1936_MOESM1_ESM.docx]

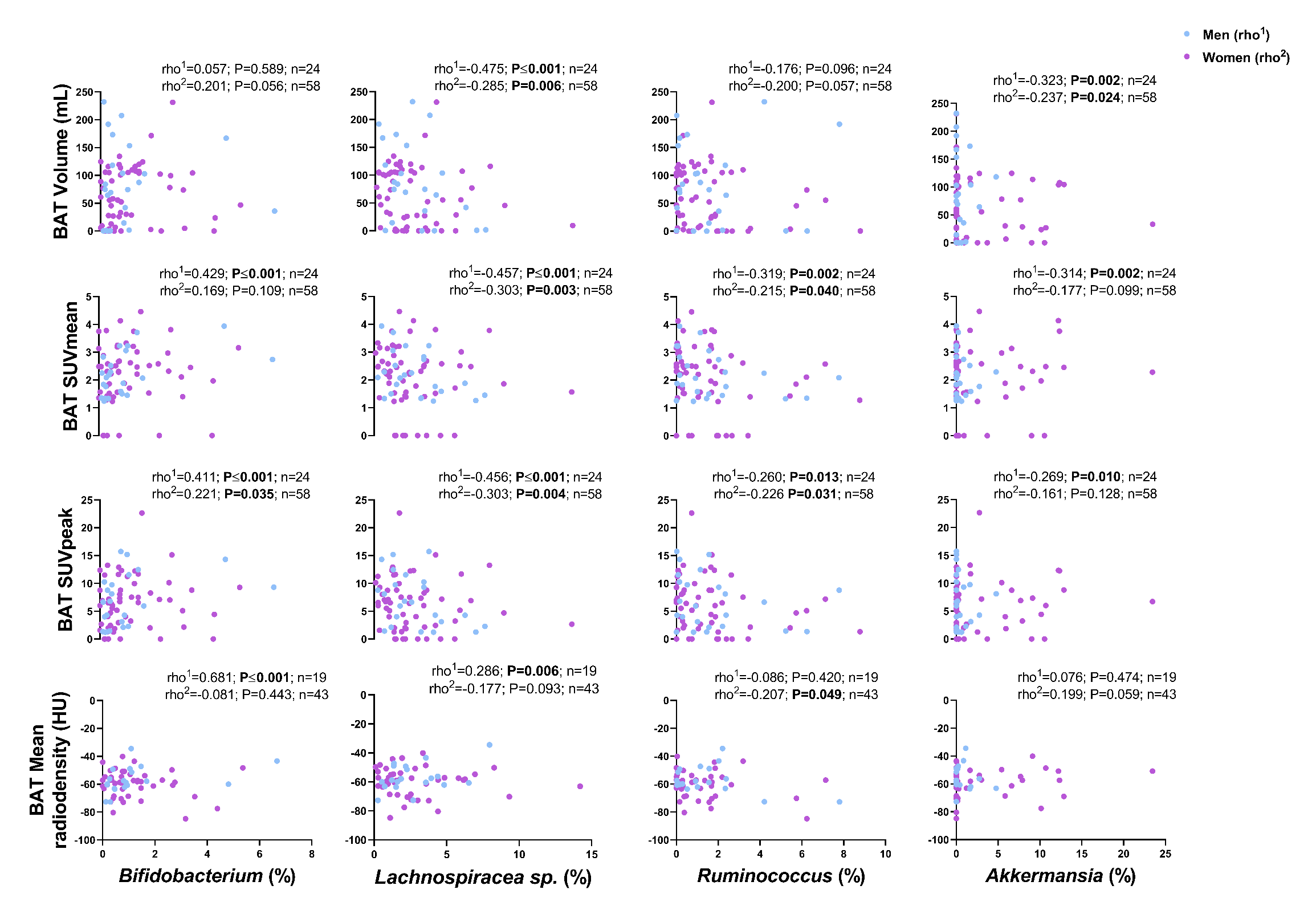


**Figure S1.** **Partial Spearman correlations of the relative abundance of *Bifidobacterium*, *Lachnospiraceae sp.*, *Ruminococcus* and *Akkermansia* genera with cold-induced BAT volume, SUVmean, SUVpeak and mean radiodensity adjusted by sex, after adjusting for the PET/CT scan date**. Blue circles represent men participants and purple circles show women participants. Rho^1^=Partial Spearman's correlations coefficient for men. Rho^2^=Partial Spearman's correlations coefficient for women. P= p-value from univariate partial Spearman correlation. N= sample size. BAT SUVmean and SUVpeak are shown relative to lean mass. BAT: brown adipose tissue; HU: Hounsfield Units; PET/CT: positron emission tomography/computed tomography; SUV: standardized uptake value.
