# Supplementary material for: Fecal microbiota composition is related to brown adipose tissue 18F-fluorodeoxyglucose uptake in young adults
Source: J Endocrinol Invest. 2022 Oct 15;46(3):567–76. doi: 10.1007/s40618-022-01936-x (PMC9938059; doi:10.1007/s40618-022-01936-x)
Supplement: Supplementary file 2 — Supplementary file2 (DOCX 16 KB) [file 40618_2022_1936_MOESM2_ESM.docx]

| **Table S1.**  Differences in PET/CT and fecal microbiota composition variables between genders. | | | |
| --- | --- | --- | --- |
|  | **Men**  **N=24** | **Women**  **N=58** |  |
|  | *Mean ± SD* | *Mean ± SD* | *p-value* |
| *PET/CT variables* |  |  |  |
| BAT volume (mL) | 83.4 ± 72.8 | 63.0 ± 53.3 | 0.370 |
| BAT SUVmean | 2.2 ± 0.8 | 2.1 ± 1.1 | 0.683 |
| BAT SUVpeak | 6.7 ± 4.8 | 6.3 ± 4.7 | 0.683 |
| BAT Mean radiodensity (HU)* | -56.8 ± 9.4 | -59.9 ± 9.8 | 0.536 |
| *Fecal microbiota variables* |  |  |  |
| *Composition (Phylum)* |  |  |  |
| *Actinobacteria* (%) | 1.4 ± 1.7 | 1.8 ± 1.6 | 0.179 |
| *Bacteroidetes* (%) | 39.9 ± 11.4 | 39.9 ± 7.8 | 0.338 |
| *Firmicutes* (%) | 50.5 ± 8.6 | 47.4 ± 10.3 | 0.093 |
| *Proteobacteria* (%) | 7.1 ± 4.2 | 6.5 ± 5.8 | 0.126 |
| *Verrucomicrobia* (%) | 0.6 ± 1.1 | 3.0 ± 4.9 | 0.112 |
| Data are presented as means ± standard deviations (SD). All SUV variables are shown relative to lean body mass. P values were obtained from Mann-Whitney test. *For BAT Mean radiodensity, data are missing for 10 participants (remaining men n= 19 and women n= 43). BAT: brown adipose tissue; BMI: body mass index; HU: Hounsfield Units; SUV: standardized uptake value. | | | |

s
